# Global Greening Major Contributed by Climate Change With More Than Two Times Rate Against the History Period During the 21th Century

**DOI:** 10.1111/gcb.70126

**Published:** 2025-03-11

**Authors:** Hao Zhang, Zengyun Hu, Xi Chen, Jianfeng Li, Qianqian Zhang, Xiaowei Zheng

**Affiliations:** ^1^ State Key Laboratory of Ecological Safety and Sustainable Development in Arid Lands, Xinjiang Institute of Ecology and Geography Chinese Academy of Sciences Urumqi Xinjiang China; ^2^ University of Chinese Academy of Sciences Beijing China; ^3^ School of Global Health, Chinese Center for Tropical Diseases Research Shanghai Jiao Tong University School of Medicine Shanghai China; ^4^ College of Geoinformatics Zhejiang University of Technology Hangzhou China; ^5^ The Chinese University of Hong Kong Hong Kong China

**Keywords:** CCHZ‐DISO, climate change, GGMAOC, global greening, LAI, long‐term projections, multimodel, spatial heterogeneity

## Abstract

Future variations of global vegetation are of paramount importance for the socio‐ecological systems. However, up to now, it is still difficult to develop an approach to project the global vegetation considering the spatial heterogeneities from vegetation, climate factors, and models. Therefore, this study first proposes a novel model framework named GGMAOC (grid‐by‐grid; multi‐algorithms; optimal combination) to construct an optimal model using six algorithms (i.e., LR: linear regression; SVR: support vector regression; RF: random forest; CNN: convolutional neural network; and LSTM: long short‐term memory; transformer) based on five climatic factors (i.e., Tmp: temperature; Pre: precipitation; ET: evapotranspiration, SM: soil moisture, and CO_2_). The optimal model is employed to project the future changes in leaf area index (LAI) for the global and four sub‐regions: the high‐latitude northern hemisphere (NH), the mid‐latitude NH, the tropics, and the mid‐latitude southern hemisphere. Our results indicate that global LAI will continue to increase, with the greening rate expanding to 2.25 times in high‐latitude NH by 2100 against the 1982–2014 period. Moreover, RF shows strong applicability in the global and NH models. In this study, we introduce an innovative model GGMAOC, which provides a new optimal model scheme for environmental and geoscientific research.

## Graphical abstract

Our study introduces GGMAOC, a novel modeling framework that constructs an optimal model for each region to predict future global vegetation changes. Using GGMAOC, we project leaf area index (LAI) trends worldwide and across four sub‐regions. Results suggest continued global greening, with the high‐latitude northern hemisphere experiencing a 2.25‐fold increase in LAI by 2100. Among the models, random forest demonstrates strong predictive performance. GGMAOC provides an advanced, data‐driven approach for environmental research, offering new insights into vegetation dynamics under climate change.

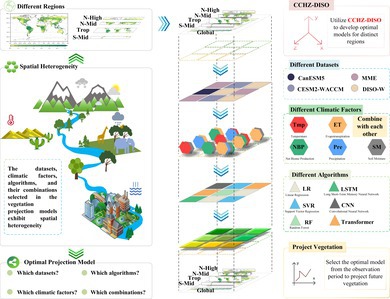

## 1 Introduction

As the primary producers within ecosystems, vegetation not only provides valuable ecosystem services such as food and fiber but also plays a critical role in the terrestrial water, carbon, and energy cycles (Piao et al. 2020; Zheng et al. 2024). Numerous studies have demonstrated that, since the 1980s, global vegetation has undergone a widespread greening trend (Chen et al. 2019; Fensholt et al. 2012; Li et al. 2024; Park et al. 2016; Zhang et al. 2024; Zhu et al. 2016). Due to the lack of sufficient observational data from field stations (Piao et al. 2020), these studies have primarily relied on satellite remote sensing‐derived vegetation indices (VIs) to examine vegetation dynamics on a large scale.

With the advancement of satellite remote sensing technology, a wide range of VIs have been developed since the 1970s. Today, several VIs have enabled continuous global vegetation monitoring since the 1980s, providing a robust foundation for studying vegetation dynamics (Zeng et al. 2022), such as the LAI, normalized difference vegetation index (NDVI), and enhanced vegetation index (EVI). Each VI has its own applicability and characteristics. For instance, NDVI is less sensitive in densely vegetated areas and is easily affected by surface conditions such as snow cover and precipitation (Pre), while EVI is commonly used in dense vegetation regions like tropical rainforests (Zeng et al. 2022; Zhang et al. 2024). LAI, on the other hand, provides a better representation of the vegetation structure and function, as it refers to half of the total leaf area per unit of ground surface area (Song et al. 2021). It has been more widely adopted in studies on vegetation change (Piao et al. 2020).

There are numerous long‐term time series of LAI remote sensing data available on a global scale. Examples include the global inventory monitoring and modeling studies (GIMMS) LAI, global land surface satellite (GLASS) LAI, and global mapping (GLOBMAP) LAI, all of which derive their data using different methodologies from the advanced very high‐resolution radiometer (AVHRR) and moderate resolution imaging spectroradiometer (MODIS) sensors (Fang et al. 2013). Zhu et al. (2016) demonstrated that, while these three LAI datasets exhibit consistency in revealing global LAI trends, there are regional discrepancies among them. These differences are primarily attributed to variations in the data production methods and the inherent differences between AVHRR and MODIS (Liu et al. 2012; Ma and Liang 2022; Zhu et al. 2016; Vermote et al. 2019).

LAI is closely linked to the ecological environment and engages in reciprocal feedback with climate at regional scales (Forzieri et al. 2017; Funk and Brown 2006; Lian et al. 2020). Vegetation changes driven by climate change may disrupt ecosystem services and even lead to biodiversity loss (Cardinale et al. 2012; Lian et al. 2021; Song et al. 2023). Studies suggest that LAI can be used to identify regions vulnerable to climate change (Mahowald et al. 2016), but the future of vegetation dynamics remains uncertain due to the impacts of global warming (Nolan et al. 2018). Therefore, projecting vegetation changes is crucial for understanding future climate scenarios (Nolan et al. 2018; Ukkola et al. 2016). Existing research primarily uses climate factors related to vegetation growth, employing various models or algorithms to predict future vegetation changes.

The climatic factors associated with vegetation growth exhibit spatial heterogeneity on a global scale. Numerous studies have confirmed that CO_2_ is the primary driver of global vegetation greening; however, 28% of this greening is attributed to climate change (Piao et al. 2020). The impact of climate change on vegetation varies by region. For instance, temperature (Tmp) is the dominant factor driving vegetation greening in the high latitudes of the NH and the Tibetan Plateau, while Pre plays a key role in vegetation greening in southern Africa (Zhu et al. 2016). However, when projecting future vegetation changes using climate variables, existing studies often overlook the spatial heterogeneity of climate–vegetation interactions. For example, Chen et al. (2021) predicted future vegetation dynamics on a global scale using Tmp and Pre, while Zhao et al. (2020) employed CO_2_, Pre, Tmp, and surface radiation. As a result, these studies fail to develop optimal prediction models based on the dominant climatic factors influencing vegetation in different regions.

In the selection of models and algorithms, early research primarily employed models based on statistical relationships. While these models are straightforward, their predictive capabilities are limited (Del Grosso et al. 2008). Additionally, many biogeochemical models and dynamic global vegetation models have been widely used (Chen et al. 2022; Rineau et al. 2019; Zhao et al. 2020). Some earth system models (ESMs) within the Coupled Model Intercomparison Project (CMIP) have also been utilized to project vegetation changes (Bonan and Doney 2018; Mahowald et al. 2016; Zhang et al. 2022). However, there are substantial discrepancies among different ESMs (Anav et al. 2013; Mahowald et al. 2016; Peng et al. 2024b; Rahimpour Asenjan et al. 2023).

Due to its robust data processing capabilities, artificial intelligence (AI) has been widely applied in geography (Bai et al. 2022; Chen et al. 2021, 2022; He et al. 2020; Nguyen‐Kim et al. 2023; Sun et al. 2023; Xiao et al. 2014). As a typical AI approach, machine learning algorithms such as LR, SVR, and RF were extensively used in earlier studies, but their capacity to handle complex data remains limited (Sun et al. 2023). In recent years, deep learning has seen rapid development, with models like CNN, LSTM, and Transformer (Krizhevsky et al. 2017; Sun et al. 2023) being employed in related research to predict future vegetation. For instance, Chen et al. (2022) and Sun et al. (2023) used LSTM to forecast future vegetation dynamics. However, these studies developed a single model for all study areas, without accounting for the spatial heterogeneity of the models.

Chen, Chen, Hu, and Zhou, distance between indices of simulation and observation (CCHZ‐DISO), based on Euclidean spatial distance, facilitates the comparison of large datasets with observational data (OBS), enabling comprehensive quantification of big data. Due to its simplicity and flexibility, CCHZ‐DISO can be applied across multiple fields and disciplines (Hu et al. 2019, 2022; Zhou et al. 2021). It has already been widely adopted in various studies (Li and Zhang 2023; Li et al. 2023; Nie et al. 2023; Peng et al. 2024a; Wang et al. 2023).

This study addresses three key questions regarding the future changes in global vegetation: (1) Do the performances of AI algorithms and the factors influencing vegetation changes exhibit spatial heterogeneity? (2) How can the most optimal models be selected for different regions? (3) What are the future trends of global vegetation?

In this study, the CCHZ‐DISO model is employed to identify the optimal model for both the global scale and four sub‐regions. The model selection process involves screening the best ESMs data from the World Climate Research Programme's (WCRP's) Coupled Model Intercomparison Project phase 6 (CMIP6), determining the dominant climatic factor combinations for each region and selecting the most suitable algorithm to predict LAI dynamics under multiple shared socioeconomic pathways (SSP126, SSP245, SSP370, SSP585) by the end of the 21st century. Additionally, using the original CMIP6 data and the established prediction models, this study develops a set of DISO‐weighted climatic data (DISO‐W) through CCHZ‐DISO, which is incorporated into the modeling process. By establishing the optimal prediction model for each region, this approach allows for more accurate forecasts of future vegetation changes and captures trends in future climate change, thereby enhancing our understanding of future global carbon, water, and energy cycles. Moreover, the methodology of model development based on CCHZ‐DISO can be applied to predictions of other geographic factors, reducing uncertainty in the forecasting process.

## 2 Data

### 2.1 LAI Datasets

In this study, we utilize three widely used long‐term time series of LAI remote sensing datasets to analyze vegetation variations, which are displayed in Table 1.

**TABLE 1:** LAI datasets.

Dataset	Satellite	Spatial resolution	Temproal resolution	Duration	Source	Reference
GIMMS LAI (Version 005)	AVHRR	0.05°	Daily	1982–2014	https://doi.org/10.7289/V5TT4P69	Vermote et al. (2019)
GLASS LAI (Version 40)	AVHRR，MODIS	0.05°	8 days	1982–2014	http://www.glass.umd.edu/Download.html	Ma and Liang (2022); Xiao et al. (2016)
GLOBMAP LAI (Version 3.0)	AVHRR，MODIS	0.07°	15 days, 8 days	1982–2014	https://zenodo.org/records/4700264	Liu et al. (2012)

For the three LAI datasets, the monthly maximum values are obtained using the maximum value composite (MVC) method. Then, the data are resampled to a 0.5° resolution using bilinear interpolation. Finally, an ensemble average of the three monthly LAI datasets was calculated to derive the final LAI dataset.

### 2.2 CMIP6 Datasets

We selected monthly scale Pre, Tmp, evapotranspiration (ET), and soil moisture (SM) data from CMIP6 to represent the climatic factors, while net biome production (NBP) was used to account for CO_2_ in projecting vegetation changes (Martín‐Gómez et al. 2023). From CMIP6, we chose ESMs that included all five variables for both historical and future projections under SSP126, SSP245, SSP370, and SSP585 scenarios. Ultimately, only two models—CanESM5 and CESM2‐WACCM—met these criteria (Swart et al. 2019; Danabasoglu 2019). Monthly cumulative values were used for Pre, ET, and NBP, while monthly averages were used for Tmp and SM. The CMIP6 datasets can be found at http://doi.org/10.22033/ESGF/CMIP6.3610 and http://doi.org/10.22033/ESGF/CMIP6.10071.

## 3 Method

### 3.1 CCHZ‐DISO

In this study, CCHZ‐DISO was first used to evaluate the simulation accuracy of the models. A smaller CCHZ‐DISO value indicates better model performance. The calculation is as follows:

(1) $$\begin{array}{c} {\text{DISO}}_{i}=\\ \sqrt{{\left({\text{nors}}_{i}^{1}-{\text{nors}}_{0}^{1}\right)}^{2}+{\left({\text{nors}}_{i}^{2}-{\text{nors}}_{0}^{2}\right)}^{2}+\ldots +{\left({\text{nors}}_{i}^{n}-{\text{nors}}_{0}^{n}\right)}^{2}} \end{array}$$

where *i* = 0, 1, …, *m* and *m* represents the total number of algorithms used for modeling. ${\text{nors}}_{i}^{1}$, ${\text{nors}}_{i}^{2}$, …, ${\text{nors}}_{i}^{n}$ are the normalized model performance metrics. In this study, we selected the coefficient of determination (*R* ^2^), mean absolute error (MAE), and root mean square error (RMSE) as the statistical indicator of model performance.

In this study,

(2) $$\begin{array}{c} {\text{DISO}}_{i}=\sqrt{{\left(R_{i}^{2}-1\right)}^{2}+{\left(\mathrm{nor}{\mathrm{MAE}}_{i}-0\right)}^{2}+{\left({\text{norRMSE}}_{i}-0\right)}^{2}}\; \end{array}$$

In addition, CCHZ‐DISO was used in this study to construct the DISO‐W. Since only two datasets from CMIP6 met the requirements of this study, there were two sets of data used for modeling and model evaluation for any given algorithm *i*. For dataset *l* within the ESMs and the model established by algorithm *i*, the model's value is obtained through Equation (2). Based on this value, we assigned equal weights to the five climatic factors within dataset *l*. The calculation of the weights is as follows:

(3) $$\begin{array}{c} w_{i}^{l}=\frac{\frac{1}{{\text{DISO}}_{i}^{l}}}{\sum_{l=1}^{2}\left(\frac{1}{{\text{DISO}}_{i}^{l}}\right)}\; \end{array}$$

where *l* = 1, 2.

Ultimately, in algorithm *i*, the two ESM datasets are multiplied by their respective weights to generate the DISO‐W. The calculation for a specific climatic factor within the DISO‐W is as follows:

(4) $$\begin{array}{c} x_{i}^{*}=\sum_{l=1}^{2}w_{i}^{l}\times x_{i}^{l}\; \end{array}$$

where *x* is a climatic factor, $x_{i}^{l}$ is a climatic factor of dataset *l*, and $x_{i}^{*}$ is a climatic factor of dataset DISO‐W.

Additionally, this study also performed an ensemble averaging of the selected two ESMs datasets, multi‐model ensemble mean dataset (MME). Therefore, a total of four climatic datasets were used in the modeling process: two ESM datasets from CMIP6, MME, and DISO‐W. Each of these datasets includes Tmp, Pre, Et, SM, and NBP.

The DISO code can be downloaded and used from the following GitHub repository: https://github.com/zhangZHUO0326/CCHZDISO.

### 3.2 Model Development

This study proposes a novel modeling approach, GGMAOC (grid‐by‐grid; multi‐algorithms; optimal combination). GGMAOC fully considers the spatial heterogeneity of data and algorithms. For different grids or regions, it selects the optimal combination of models and algorithms based on CCHZ‐DISO, choosing the model with the minimum DISO value as the optimal predictive model for that specific grid or region.

Taking the vegetation prediction model established in this study as an example, the specific steps of GGMAOC are as follows:

Step 1: Treating the entire globe as a single grid (region).

Step 2: The selected climate factors include Tmp, Pre, ET, SM, and CO_2_. The five factors can form 31 possible combinations.

Step 3: In this study, the available CMIP6 datasets include only two options, CanESM5 and CESM2‐WACCM. To reduce uncertainties in the modeling process, we also established two additional datasets using MME and DISO‐W methods. As a result, this study includes a total of four selectable datasets.

The data selection includes 31 climate factor combinations and 4 datasets, resulting in a total of 31 × 4 = 124 combinations.

Step 4: We selected six algorithms, including LR, SVR, RF, CNN, LSTM, and Transformer.

Step 5: Considering the combinations of data and algorithms, there are a total of 124 × 6 = 744 possible combinations, meaning that 744 models can be established for the global study area.

Step 6: Finally, CCHZ‐DISO is applied to evaluate all 744 models, and the model with the lowest CCHZ‐DISO value is identified as the optimal model.

Step 7: If there are multiple study regions, such as in this study where the global area is divided into four subregions based on latitude, then Steps 1 to 6 are repeated for each region to select the optimal model.

GGMAOC is not only applicable to LAI prediction but can also be utilized in predictive modeling across various disciplines, providing a flexible and adaptive framework for multi‐model optimization.

**FIGURE 1:**
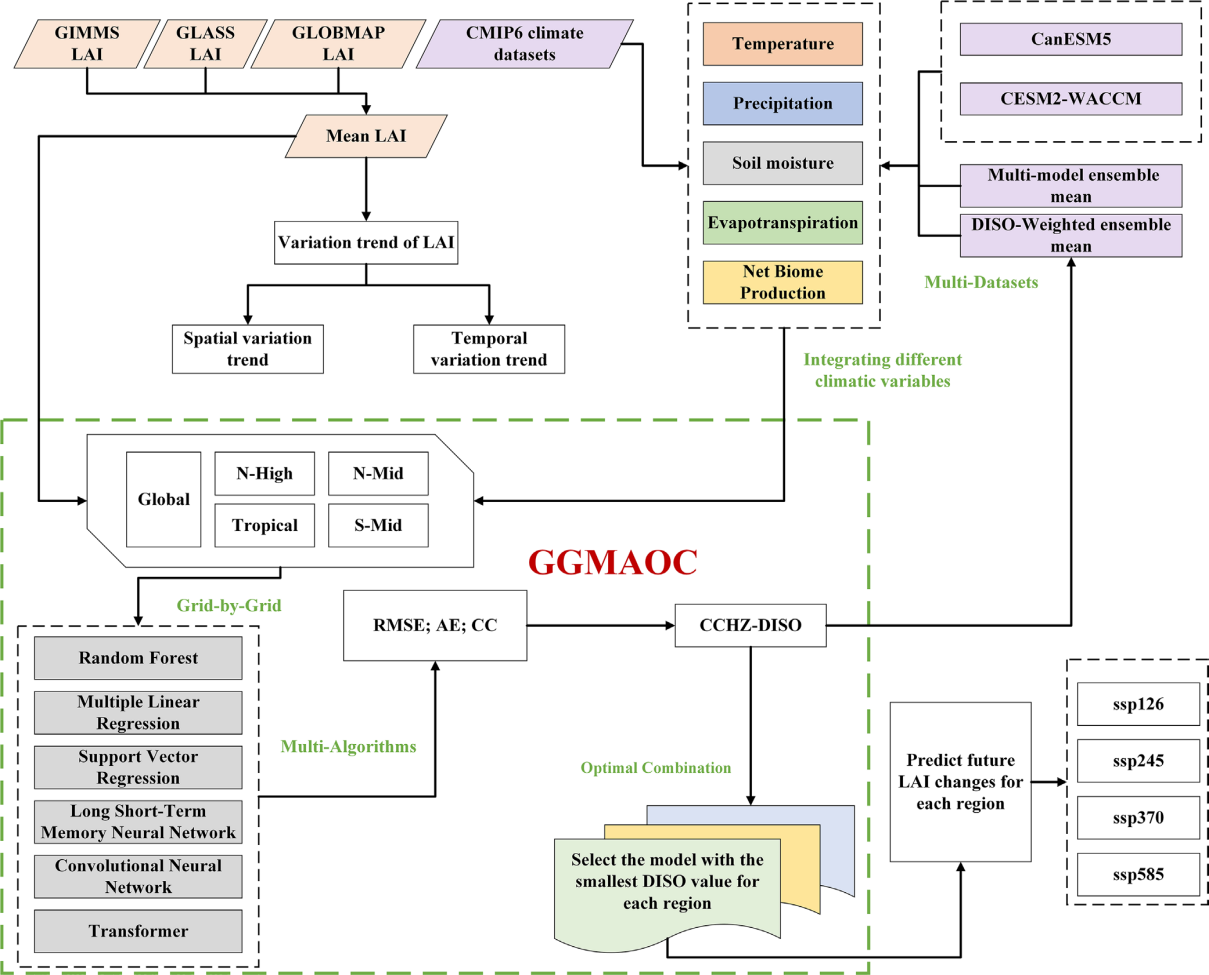
The technical workflow of the study.

Considering that vegetation trends and mechanisms differ across latitudinal zones (Zhu et al. 2016), this study divides the globe into five study regions: the global scale (Global), the high‐latitude NH (60° N–90° N) (N‐High), the mid‐latitude NH (30° N–60° N) (N‐Mid), the tropics (latitudes below 30°) (Trop), and the mid‐latitude Southern Hemisphere (30° S–60° S) (S‐Mid). For each region, GGMAOC was used to select the optimal model for projecting vegetation changes from various algorithm and climatic factor combinations based on CCHZ‐DISO. The technical workflow of the study is illustrated in Figure 1.

**FIGURE 2:**
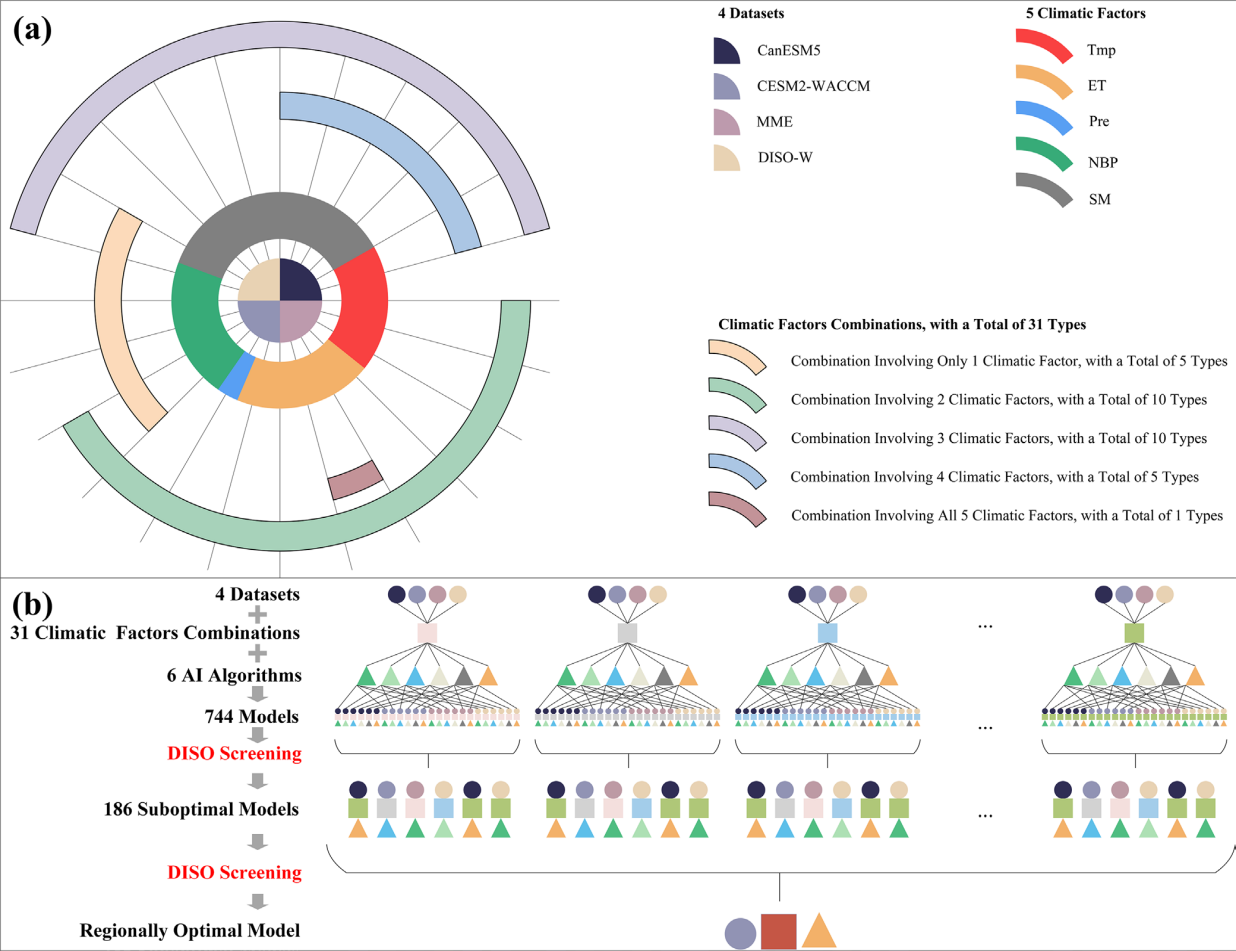
Schematic of modeling for a specific region. (a) illustrates the combination of five climatic factors in the four datasets, with each dataset containing 31 possible combinations. (b) depicts the process of selecting the optimal model for the region from 744 models (constructed from four datasets, 31 climatic factor combinations, and six algorithms) using the DISO (distance between indices of simulation and observation) evaluation method.

In this study, for each of the four selected climatic datasets, there are five climatic factors included in each dataset. As a result, there are a total of 31 possible combinations of climatic factors within each dataset (Table 2, Figure 2a). Each of these combinations is paired with six different algorithms for modeling, allowing for 744 models to be constructed within each region. Based on this, for each climatic factor combination, DISO is used to select the best‐performing model, resulting in 31 optimal models across all combinations. For each region, the study considers the optimal algorithm, dataset, and climatic factor combination. DISO is then applied to evaluate the 31 optimal models, and the model with the smallest DISO value—that is, the best‐performing model—is ultimately selected to predict LAI under different emission scenarios for that region (Figure 2b).

**TABLE 2:** Combinations of climatic factors.

Number	Climate variables				
1	ET				
2		SM			
3			Pre		
4				Tmp	
5					CO_2_
6	ET	SM			
7	ET		Pre		
8	ET			Tmp	
9	ET				CO_2_
10		SM	Pre		
11		SM		Tmp	
12		SM			CO_2_
13			Pre	Tmp	
14			Pre		CO_2_
15				Tmp	CO_2_
16	ET	SM	Pre		
17	ET	SM		Tmp	
18	ET	SM			CO_2_
19	ET		Pre	Tmp	
20	ET		Pre		CO_2_
21	ET			Tmp	CO_2_
22		SM	Pre	Tmp	
23		SM	Pre		CO_2_
24		SM		Tmp	CO_2_
25			Pre	Tmp	CO_2_
26	ET	SM	Pre	Tmp	
27	ET	SM	Pre		CO_2_
28	ET	SM		Tmp	CO_2_
29	ET		Pre	Tmp	CO_2_
30		SM	Pre	Tmp	CO_2_
31	ET	SM	Pre	Tmp	CO_2_

## 4 Results

### 4.1 Global Vegetation Variations From 1982 to 2014

**FIGURE 3:**
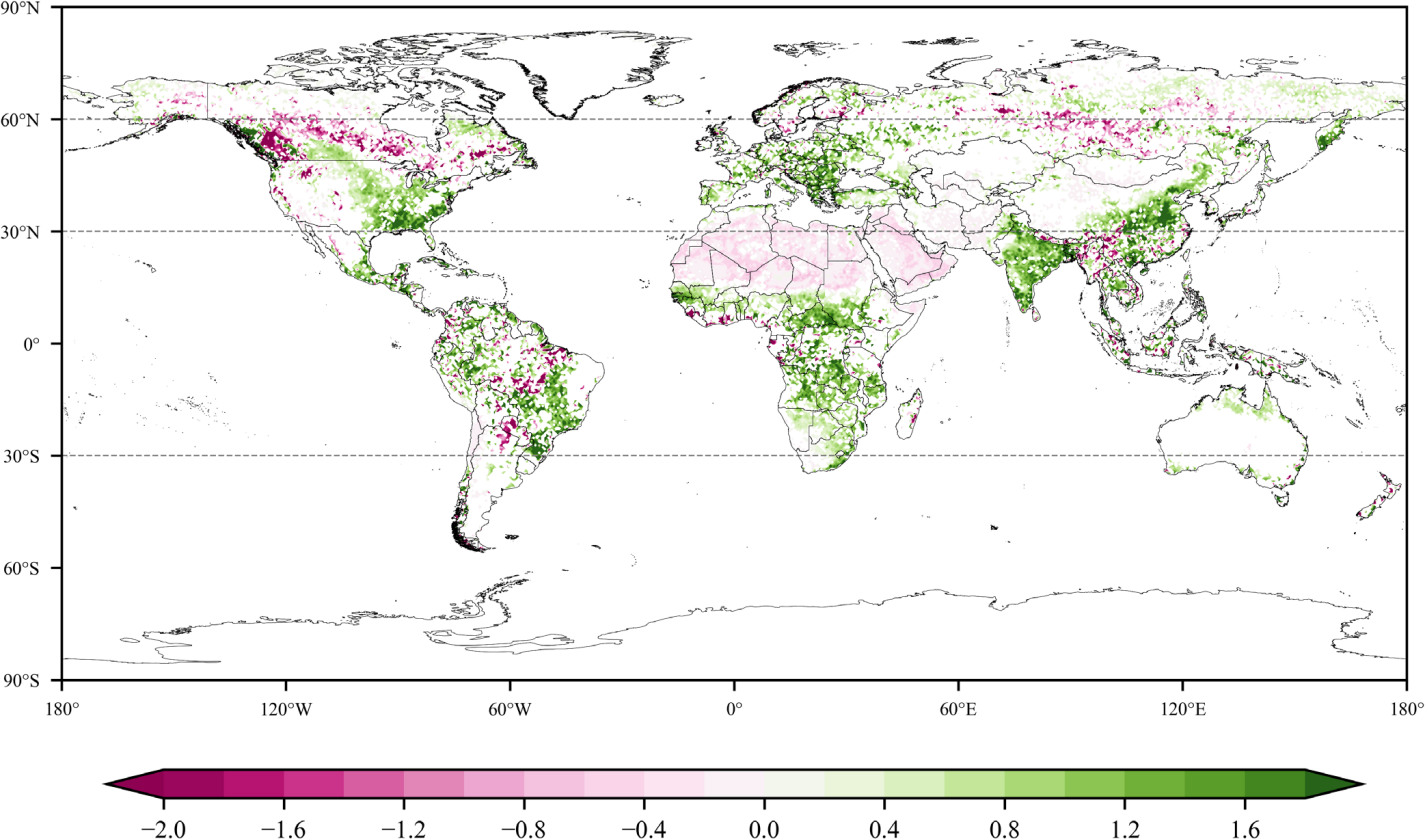
Significant global LAI (leaf area index) trends from 1982 to 2014 (*p* < 0.05), unit: 10^−2^ m^2^ m^−2^ year^−1^. Map lines delineate study areas and do not necessarily depict accepted national boundaries.

Using the Mann‐Kendall (M‐K) trend analysis (Mann 1945), we examined global vegetation change trends from 1982 to 2014 (Figure 3). During this period, 52.16% of the vegetated area experienced a greening trend, with 32.41% showing significant greening. The most prominent greening occurred in regions such as China, India, the eastern United States, Europe, the eastern South America, and southern Africa. In contrast, browning trends were observed in central Russia, Canada, central South America, Southeast Asia, and across northern Africa.

**FIGURE 4:**
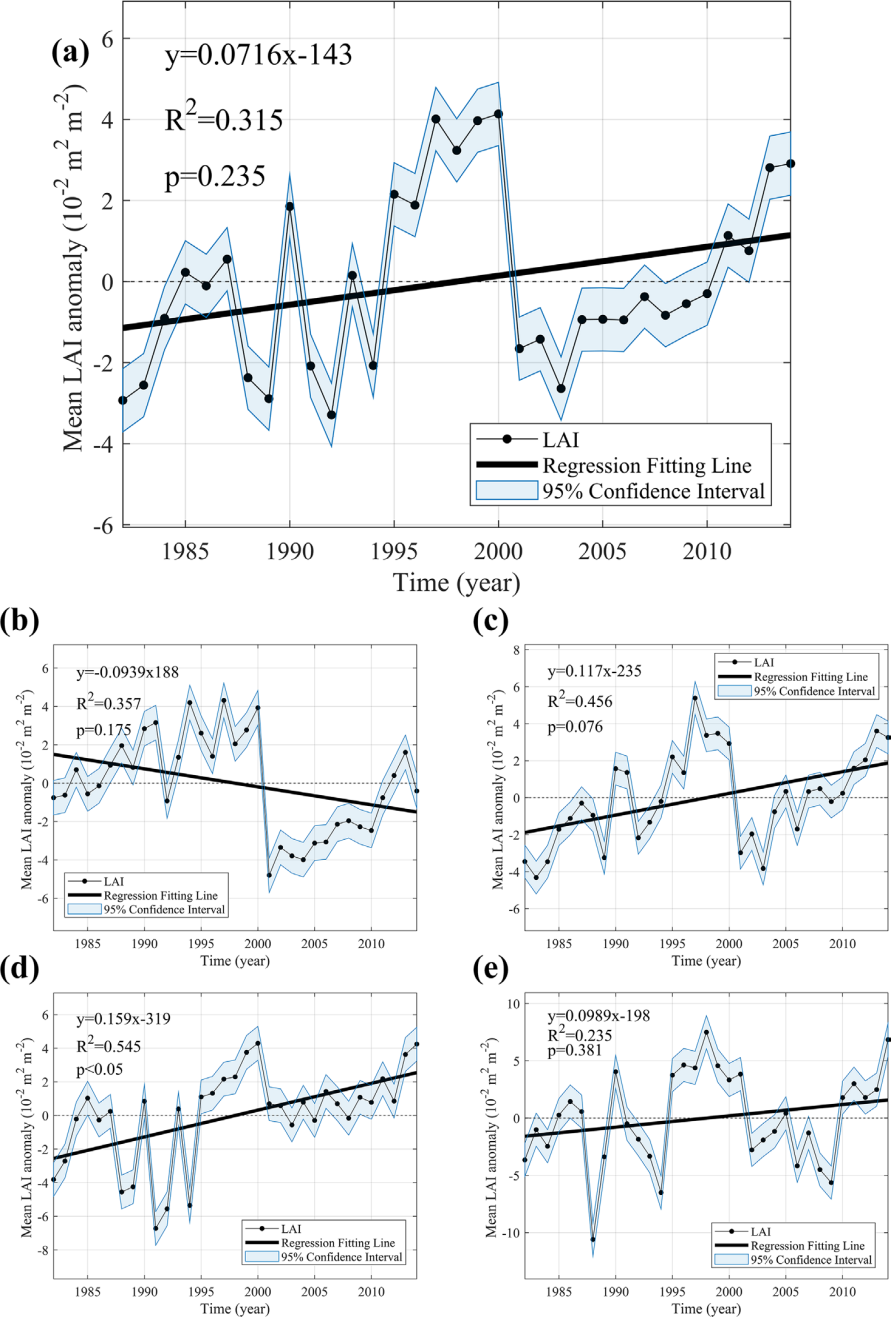
LAI (leaf area index) trends from 1982 to 2014 by the region. (a–e) represents Global, N‐High (60° N–90° N), N‐Mid (30° N–60° N), Trop (latitudes below 30°), and S‐Mid (30° S–60° S), respectively.

Additionally, we analyzed the LAI change trends for each region from 1982 to 2014 (Figure 4). Over the study period, the global LAI increased at a rate of 0.072 m^2^ m^−2^ year^−1^. Except for the N‐High region, all other regions exhibited greening trends. The Tropics showed the most pronounced greening, with a significant increase at a rate of 0.159 m^2^ m^−2^ year^−1^. N‐Mid followed this, with a greening rate of 0.117 m^2^ m^−2^ year^−1^, while S‐Mid showed a modest greening rate of 0.01 m^2^ m^−2^ year^−1^. In contrast, N‐High exhibited an LAI change rate of −0.1 m^2^ m^−2^ year^−1^ between 1982 and 2014. However, this region displayed greening trends during the periods 1982–2000 and 2001–2014, with browning occurring only in 2000–2001.

### 4.2 Selection of the Optimal Model According to the Climate Factors, CMIP 6 Datasets, and AI Algorithms

**FIGURE 5:**
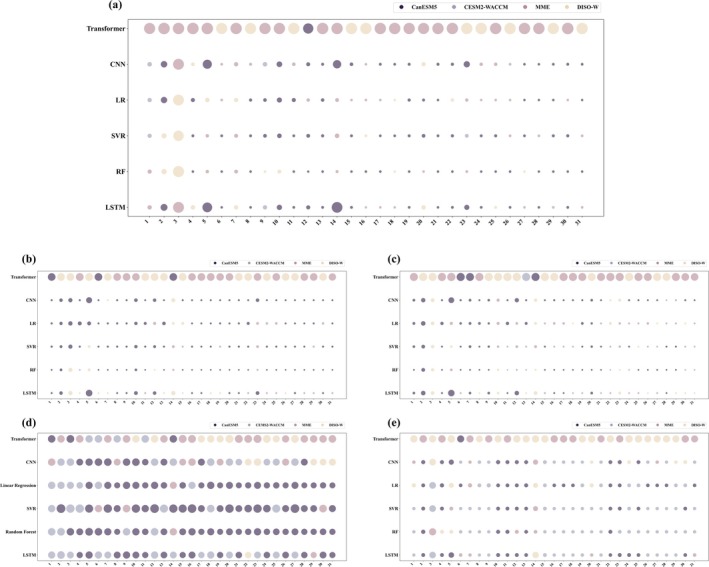
Panels (a–e) represent the DISO (distance between indices of simulation and observation) values of the 186 models constructed with the optimal dataset in each region—Global, N‐High (60° N–90° N), N‐Mid (30° N–60° N), Trop (latitudes below 30°), and S‐Mid (30° S–60° S). The size of each bubble indicates the DISO value, with smaller bubbles representing lower DISO values and, thus, better‐performing models.

For each region, this study utilized four datasets—CanESM5, CESM2‐WACCM, MME, and DISO‐W—to construct models. The CCHZ‐DISO method was applied initially to select the optimal datasets. For any given region, each climatic factor combination *i* paired with algorithm *Ai* and matched across the four datasets resulted in four models. CCHZ‐DISO was then used to identify the optimal model among these four. With 31 climatic factor combinations and six algorithms applied, a total of 186 models could be selected for each region (Figure 5). In the Global, N‐High, and Trop regions, the optimal models were derived from CanESM5, while in the N‐Mid and S‐Mid regions, CESM2‐WACCM produced the best models.

**FIGURE 6:**
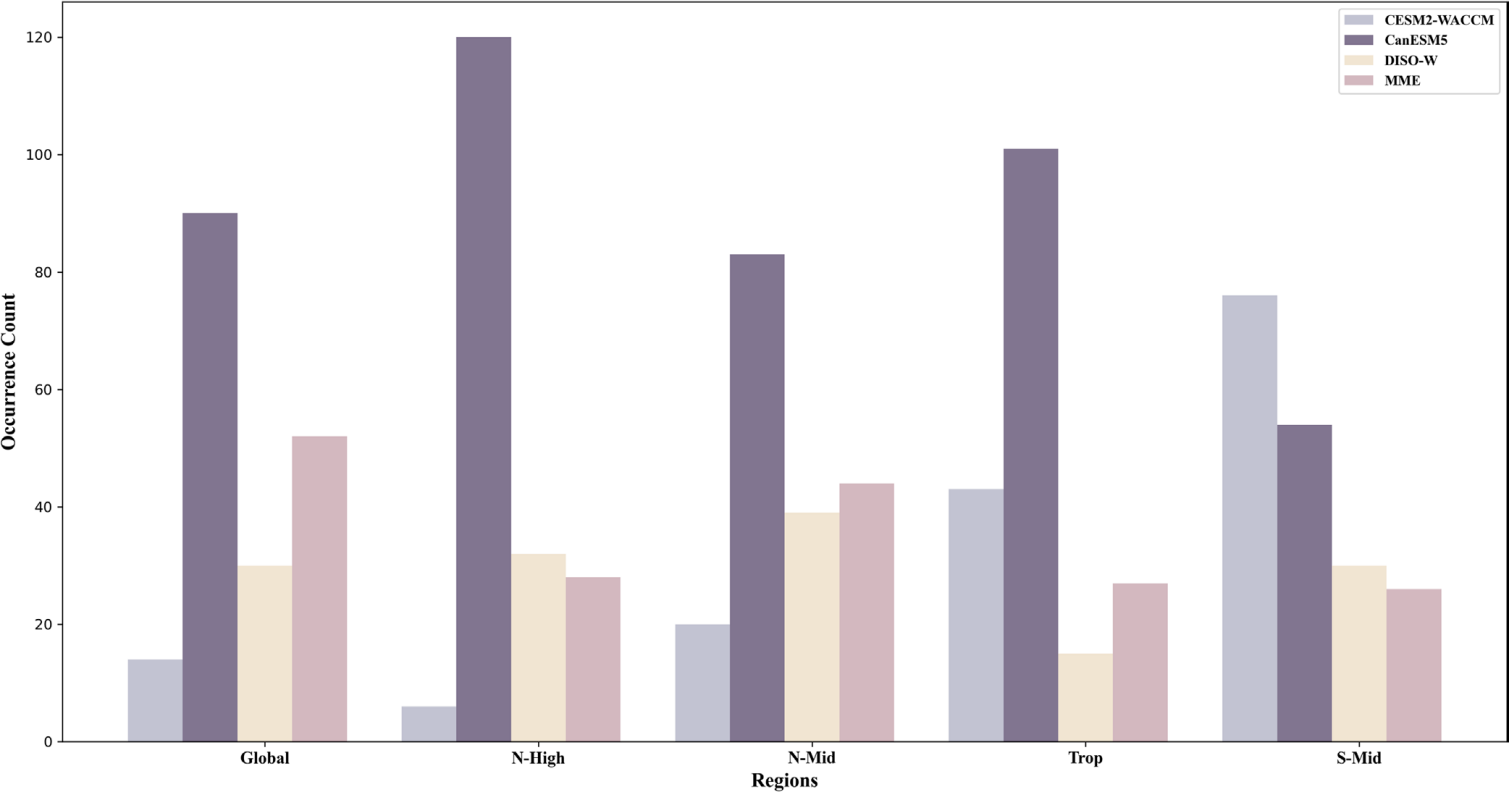
The number of models involving each dataset across the 186 models in each region.

Based on this analysis, we examined the frequency with which each dataset contributed to the 186 models in each region (Figure 6). In the Global, N‐High, N‐Mid, and Trop regions, models involving the CanESM5 dataset had the highest frequency, appearing 90, 120, 83, and 101 times, respectively. While CESM2‐WACCM had the highest frequency in the S‐Mid region, appearing 76 times, it showed the lowest frequencies in the Global, N‐High, and N‐Mid regions, with particularly low representation in N‐High, where it appeared only six times.

**FIGURE 7:**
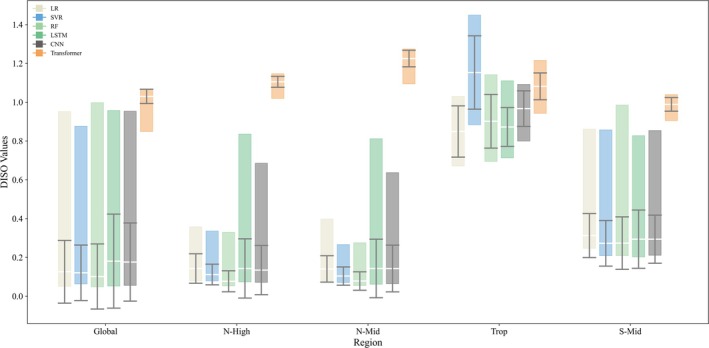
Statistical summary of DISO (distance between indices of simulation and observation) values for models constructed with each algorithm across the 186 models in each region. The upper boundary of each box represents the maximum DISO value, and the lower boundary represents the minimum DISO value. The mean DISO value for all models is indicated by a white line, while the whiskers represent the standard deviation, reflecting the level of uncertainty associated with models constructed by each algorithm.

Building on the 186 preliminary models selected for each region, this study further analyzed the usage of each algorithm (Figure 7). In the Global and N‐High regions, the optimal predictive models were constructed with RF, which also demonstrated the lowest mean DISO values and the smallest uncertainties in these regions. The optimal models for S‐Mid and Trop were built with LSTM and LR, respectively. Across all regions except Trop, LSTM‐based models exhibited high uncertainty, whereas SVR showed the opposite trend, with low uncertainty in all regions except Trop. The models constructed with CNN underperformed across all regions, and the models built with Transformer exhibited the poorest performance in every region.

**FIGURE 8:**
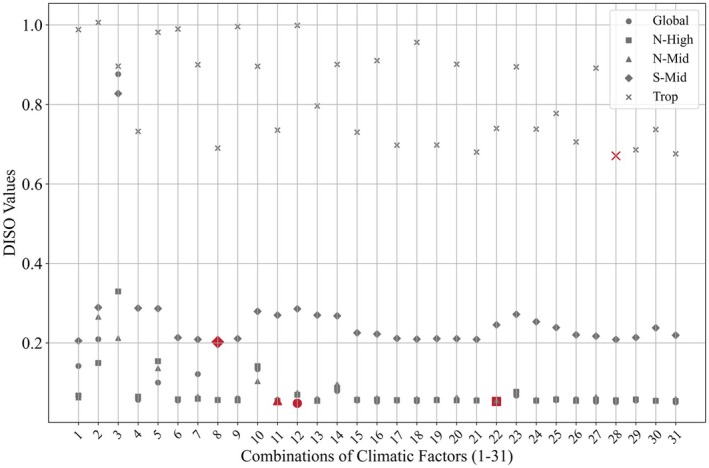
DISO (distance between indices of simulation and observation) values for the optimal models constructed with 31 combinations of climatic factors (Table 2) in each region. The red color indicates the model with the smallest DISO value in each region, representing the regional optimal model.

Based on the 186 selected models for each region, we further identified the optimal model for each combination of climatic factors, yielding a total of 31 models per region (Figure 8). In the Global region, models involving the SM and CO_2_ combination were optimal, while in N‐High, the SM, Pre, and Tmp combination yielded the best models. In N‐Mid, models with the SM and Tmp combination were most effective, whereas in S‐Mid, the optimal models incorporated ET and Tmp. For Trop, models involving ET, SM, Tmp, and CO_2_ were optimal. Additionally, for both the Global and N‐High regions, the DISO values of models built with combinations 15–31 remained stable, suggesting that models with three or more climatic factors demonstrate higher suitability and stability in these regions.

### 4.3 Projection of the Future LAI Variations Based on the Optimal Models

**FIGURE 9:**
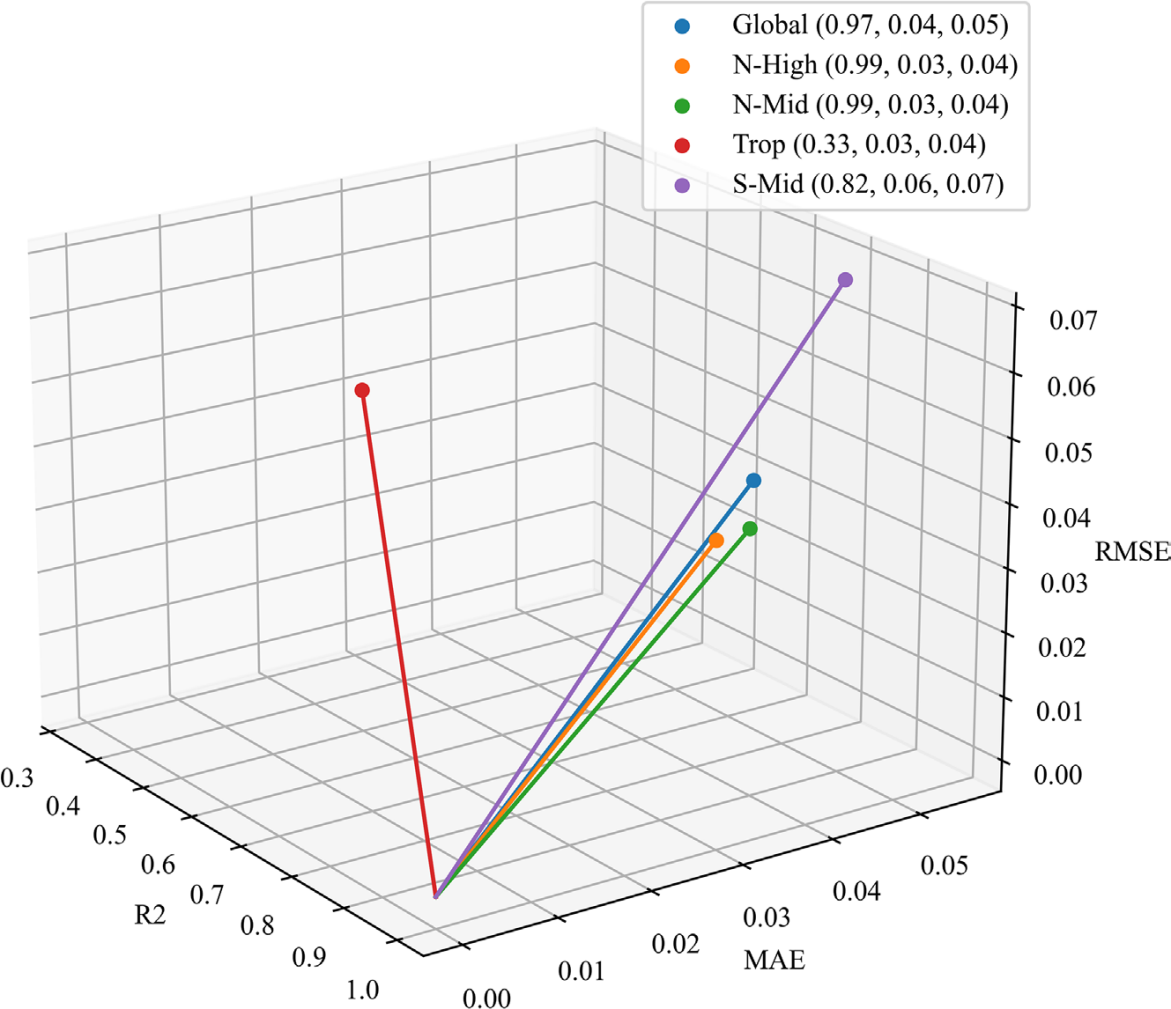
DISO (distance between indices of simulation and observation) values of optimal models for each region.

Following the approach of selecting the best‐matched dataset, optimal climatic factor combination, and most suitable algorithm for each region, this study used CCH‐DISO to filter the optimal model from the 744 models constructed for each region. The datasets and algorithms used for each optimal model are listed in Table 3, while the DISO values for each algorithm are shown in Figure 9. The fit of the models built by each algorithm is illustrated in Figure S2.

**TABLE 3:** Algorithms and datasets used in optimal models for each region.

Region	DISO_value	Dataset	Algorithm	Climatic variable combination				
Global	0.05	CanESM5	RF		SM			CO_2_
N‐High	0.05	CanESM5	RF		SM	Pre	Tmp	
N‐Mid	0.05	CESM2‐WACCM	RF		SM		Tmp	
S‐Mid	0.20	CESM2‐WACCM	LSTM	ET			Tmp	
Trop	0.67	CanESM5	LR	ET	SM		Tmp	CO_2_

In the Global region, models that included SM and CO_2_ demonstrated the best performance. Tmp emerged as a key factor in the optimal models for both the Northern and Southern Hemispheres, and, except for S‐Mid, SM was included in modeling for all regions. ET contributed to the optimal models only in the Trop and S‐Mid regions, while Pre was included solely in the N‐High region. Tmp significantly influences vegetation across all regions, SM has a major impact on vegetation in the NH, and ET plays a dominant role in the Trop and S‐Mid regions. Notably, the optimal models in each region do not include all climatic factors; instead, models with 2–4 climatic factors are found to be the most effective.

**FIGURE 10:**
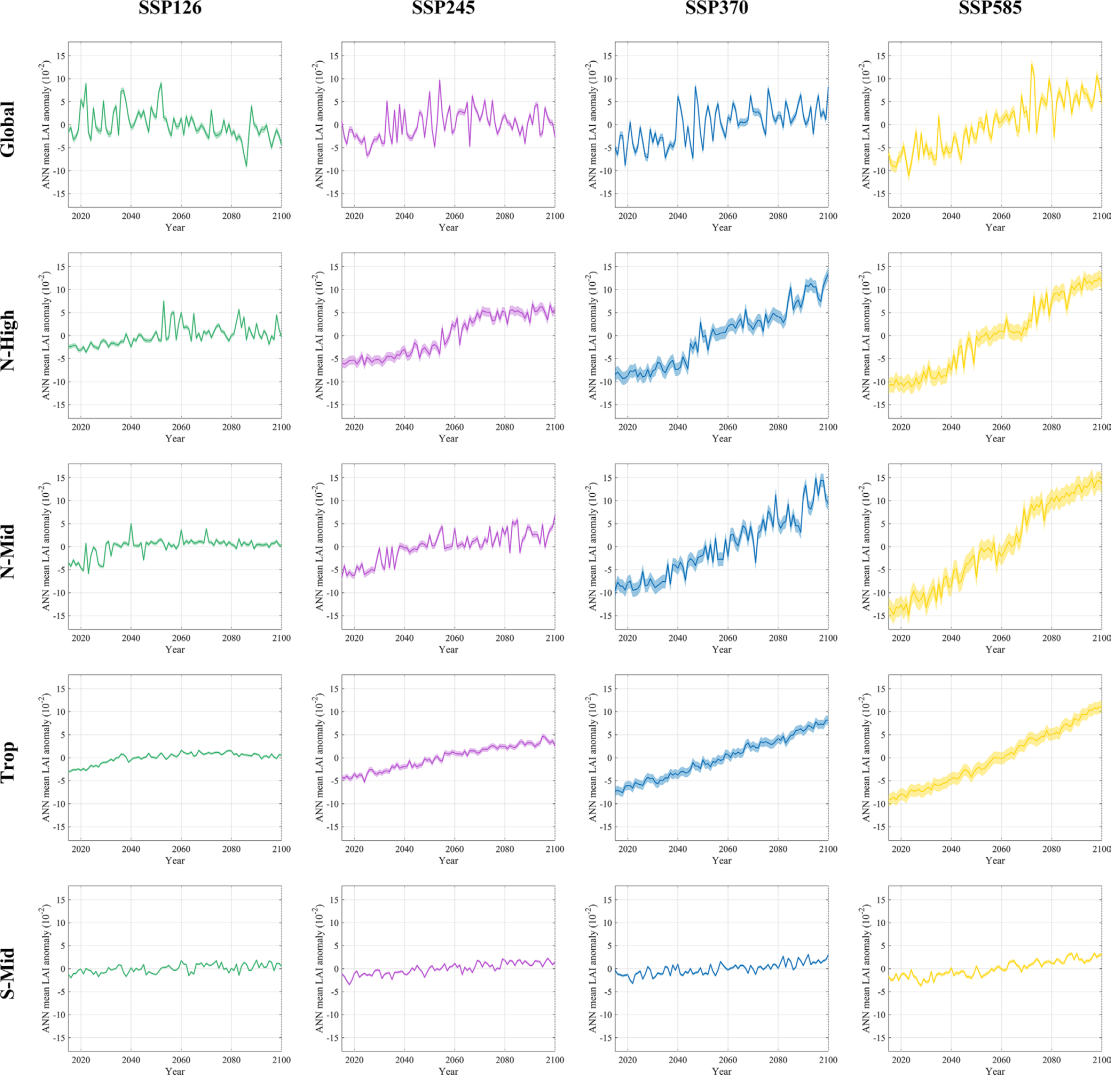
Future trends in LAI (Leaf Area Index) across different regions under various emission scenarios.

Based on the optimal models for each region, we projected the trends in LAI changes under different emission scenarios by the end of this century (Figure 10), with the corresponding rates of change presented in Table 4. Across all regions, the greening trend in LAI gradually intensifies from SSP126 to SSP585. In the SSP126 scenario, the Global region initially exhibits a significant browning trend, but after 2081, it shifts toward greening. Under other emission scenarios, all regions show a significant overall increase in LAI.

**TABLE 4:** Future LAI change rates across different regions under various emission scenarios. Unit: ×10^−3^.

Region	Year	SSP126	SSP245	SSP370	SSP585
Global	2015–2100	−0.52*	0.46*	1.03*	1.99*
	2021–2040	0.67	3.16*	0.78	0.99
	2041–2060	−0.67	1.67	0.53	3.00*
	2061–2080	−0.71	−1.13	0.29	3.25*
	2081–2100	0.30	0.40	0.96	1.82
N‐High	2015–2100	0.47*	1.70*	2.52*	3.05*
	2021–2040	0.83*	1.05*	0.98*	1.68*
	2041–2060	2.47*	2.69*	3.95*	3.33*
	2061–2080	−0.63	1.94*	0.90	3.87*
	2081–2100	−1.18	0.54	3.92*	2.16*
N‐Mid	2015–2100	0.40*	1.08*	2.60*	3.74*
	2021–2040	2.97*	2.62*	2.04*	2.78*
	2041–2060	0.59	1.35*	1.95*	3.02*
	2061–2080	−0.14	1.23*	2.99*	6.18*
	2081–2100	0.02	0.45	4.59*	2.29*
Tropical	2015–2100	0.35*	1.03*	1.81*	2.45*
	2021–2040	1.49*	1.28*	1.32*	1.68*
	2041–2060	0.42*	1.61*	1.86*	2.45*
	2061–2080	0.20	1.01*	1.72*	2.82*
	2081–2100	−0.22	0.67*	2.28*	3.29*
S‐Mid	2015–2100	0.21*	0.42*	0.40*	0.63*
	2021–2040	0.30	0.13	1.21*	0.58
	2041–2060	0.54	0.68*	0.74*	0.65*
	2061–2080	0.06	0.62	0.09	0.55
	2081–2100	0.46	0.02	0.37	0.55

* *p* < 0.05.

In the future, the NH is projected to become a hotspot for greening. Under the SSP126 and SSP245 scenarios, the N‐High region shows the highest greening rates, at 0.047 and 0.17 × 10^−2^ m^2^ m^−2^ year^−1^, respectively. However, under the SSP370 and SSP585 scenarios, N‐Mid exhibits the highest greening rates, at 0.26 and 0.34 × 10^−2^ m^2^ m^−2^ year^−1^, respectively. This indicates that under low‐emission scenarios, N‐High serves as the key region for vegetation growth, while under high‐emission scenarios, N‐Mid emerges as the hotspot for greening. Additionally, the S‐Mid region shows the smallest changes in LAI compared to other regions, with emission scenarios having a minimal impact on its rate of change. Under SSP245 and SSP370, the greening rates are nearly identical, at 0.04 × 10^−2^ m^2^ m^−2^ year^−1^.

Across all regions, a deceleration in greening—and even browning—appears after 2061 under different emission scenarios. For instance, in the Global region under SSP126, SSP245, and SSP370, browning intensifies after 2061, followed by a return to greening after 2081. Under SSP585, however, the slowdown in greening does not occur until 2081. Under SSP245, all regions experience a reduction in greening rates from 2061 to 2100. Similarly, under SSP370, greening slows across all regions between 2061 and 2080, but from 2081 to 2100, greening accelerates again. This emphasizes the persistent uncertainty in future vegetation changes across different regions.

Additionally, we calculated the changes in greening rates from 2015 to 2100 relative to the 1982–2014 period (Table 5). As radiative forcing levels increase, the greening rate also rises. Under different SSP scenarios, the global greening rate relative to 1982–2014 ranges from −172% to 178%, indicating that the greening rate may reach −1.72 to 1.78 times the 1982–2014 levels. The rate changes for N‐High, N‐Mid, Trop, and S‐Mid are −49% to 225%, −65% to 219%, −78% to 54%, and −79% to −36%, respectively.

**TABLE 5:** Percentage change in greening rate in the future (2015–2100) relative to 1982–2014. Unit: %.

Region	SSP126	SSP245	SSP370	SSP585
Global	−172.10	−35.56	43.86	178.16
N‐High	−49.51	81.42	168.31	225.32
N‐Mid	−65.64	−7.87	121.98	219.88
Tropical	−78.09	−35.08	13.97	54.04
S‐Mid	−79.05	−57.64	−59.93	−36.20

Among these regions, N‐High shows the largest change in the greening rate across all SSP scenarios. By the end of the 21st century, the LAI greening rate in the N‐High region is projected to reach −0.49 to 2.25 times the 1982–2014 levels. While N‐High exhibits the highest greening rate under low‐emission scenarios and N‐Mid leads under high‐emission scenarios (Table 4), N‐High consistently demonstrates the largest increase in greening rate across all SSPs.

## 5 Discussion

### 5.1 DISO‐W Should Be Widely Applied for Multiple Models

Generally, MME is widely used to reduce the uncertainties for multiple models due to the differences among ESMs (Cai et al. 2021; Zhu et al. 2016). However, MME assigns equal weights to all ESMs, which fail to capture the strengths of individual models (Zhu et al. 2018). The “hot model” issue in CMIP6 highlights the need to assign greater weight to better‐performing models rather than relying solely on ensemble averaging (Brunner et al. 2020; Hausfather et al. 2022; Massoud et al. 2023).

**FIGURE 11:**
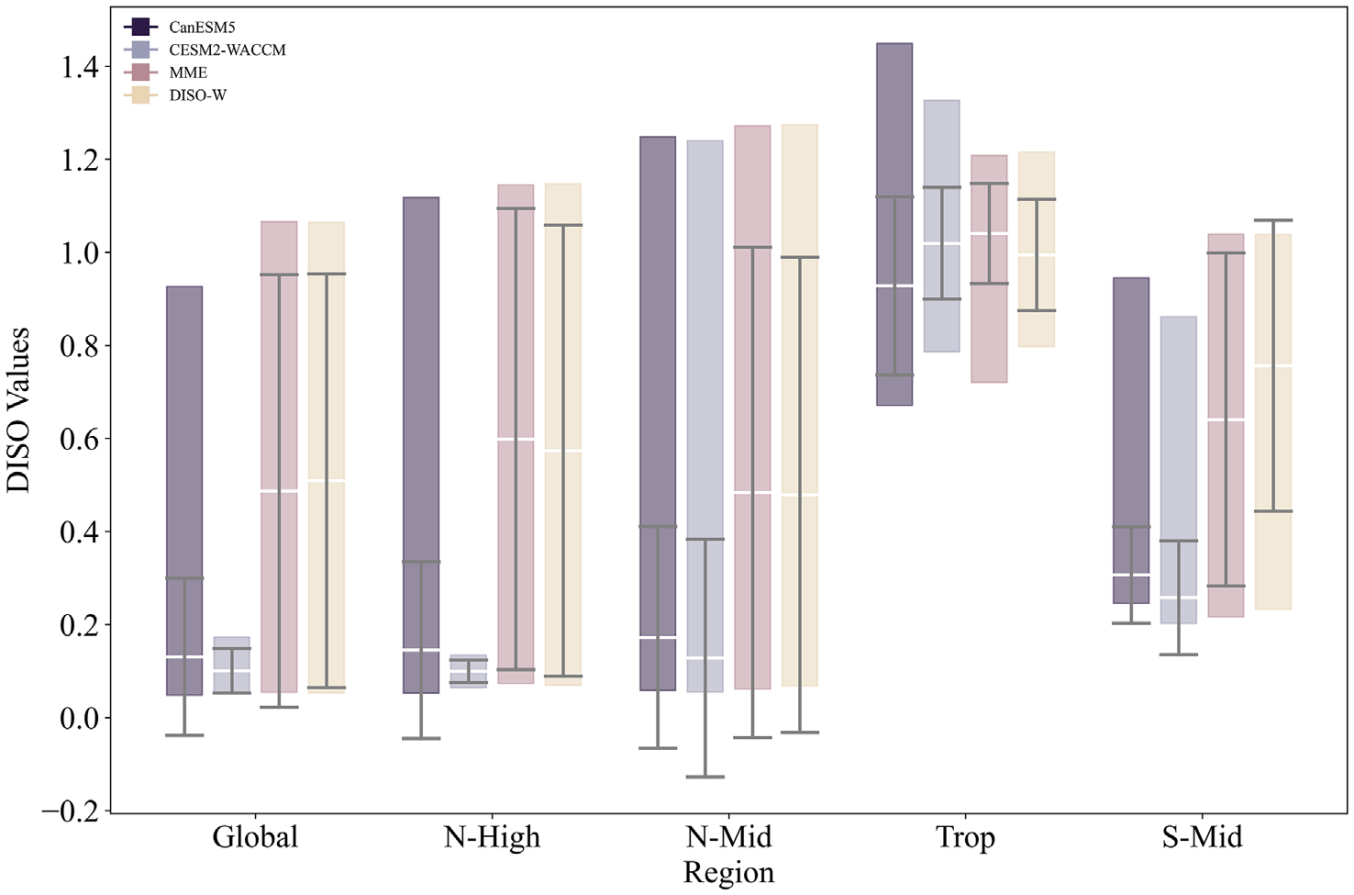
Statistical summary of DISO (distance between indices of simulation and observation) values for models constructed with each dataset across the 186 models in each region. The upper boundary of each box represents the maximum DISO value, and the lower boundary represents the minimum DISO value. The mean DISO value for all models is indicated by a white line, while the whiskers represent the standard deviation, reflecting the level of uncertainty associated with models constructed by each dataset.

Therefore, in this study, we developed a DISO‐W dataset against the MME dataset to address this issue. As shown in Figure 6, compared to the other two datasets, models constructed with MME and DISO‐W are relatively fewer across all regions. With the exception of the Global and Trop regions, the number of models involving MME and DISO‐W is similar across regions. Among the 186 models constructed from each dataset in each region, models built with MME and DISO‐W generally exhibit comparable performance across regions except in Trop, with similar minimum and mean DISO values. However, models constructed with DISO‐W exhibit reduced uncertainty in DISO values, indicating that models built with DISO‐W have lower uncertainty than those built with MME. Overall, models constructed with DISO‐W demonstrate superior performance (Figure 11).

In constructing the DISO‐W dataset, the strengths of the ESMs are extracted and integrated, while the weaknesses are minimized based on their DISO values. In contrast, the MME dataset assigns equal weights to each model, which can introduce significant uncertainties (or disadvantages) from certain models. Therefore, the DISO‐W dataset offers an improved approach for applying multiple models, effectively reducing uncertainties across different models.

### 5.2 Importance of Selecting Optimal Models for Different Regions

Different models, particularly when applied to various regions, exhibit considerable uncertainty and variability in future predictions (Jones et al. 2023; Mahowald et al. 2016; Rahimpour Asenjan et al. 2023). Previous studies have addressed this issue by selecting regionally optimal models to reduce prediction uncertainty, which aligns with the approach taken in this study (Cox et al. 2013; Hoffman et al. 2014). Comparing the datasets, climatic factor combinations, and algorithms chosen for each region highlight the importance of selecting optimal models specific to each region.

In this study, the optimal models for the Global, N‐High, and Trop regions involved the CanESM5 dataset, while the N‐Mid and S‐Mid regions used models involving the CESM2‐WACCM dataset. This indicates that the optimal models for different regions are not all built with the same dataset. Furthermore, as shown in Figure 5, the DISO values of models built with different datasets vary significantly across regions. This highlights the necessity of selecting region‐specific datasets to build the most accurate predictive models for each region.

As shown in Figure 5, the stability of the models improves as the number of climatic factors included in the models increases. However, Table 3 reveals that the optimal models for each region do not involve all the climatic factors. Instead, the optimal models include only 2–4 climatic factors. This underscores the importance of selecting regionally optimal combinations of climatic factors when building predictive models.

Table 3 shows that the optimal models for each region do not exclusively use deep learning algorithms. Only the S‐Mid region uses LSTM. In contrast, for the Global and NH regions, where model performance is relatively good, the optimal algorithm is RF. In the Trop region, where all models performed poorly, the algorithm used was LR.

Although recent studies have employed deep learning to predict LAI globally (Chen et al. 2021, 2022; Sun et al. 2023), as shown in Figure 7, the DISO values of models built with different algorithms vary significantly across regions.

Additionally, as shown in Figures 5, 7, and 8, all models perform best in the Global and N‐High regions while exhibiting the poorest performance in the Trop region. The S‐Mid region performs slightly better than the Trop region. The poor performance of models in the Trop region may be attributed to the high uncertainty caused by the saturation effect of greenness indices in dense vegetation areas (Piao et al. 2020). Therefore, selecting the optimal algorithm for each region can effectively prevent the poor model performance that results from applying a single algorithm across all regions. By using DISO to select the optimal model for each region from multiple models, we significantly reduce the uncertainties introduced by data and algorithms, enabling more accurate predictions of LAI.

### 5.3 Reliability of the Optimal Models' Projections

Regarding data selection, we identified and used the most influential combinations of climatic factors for modeling in different regions. For the Global region, we selected SM and CO_2_ as the key factors. Previous studies have shown that global vegetation changes are primarily driven by CO_2_ (Piao et al. 2020; Zhu et al. 2016). Furthermore, other research suggests that while CO_2_ is the direct cause of global vegetation changes, SM plays an indirect yet critical role in influencing these changes (Chen et al. 2023).

For the N‐High region, we selected SM, Pre, and Tmp for modeling. Zhu et al. (2016) demonstrated that in N‐High, Tmp, Pre, and radiation are the dominant factors influencing vegetation changes. Chen et al. (2023) further confirmed that Tmp is a key driver of global vegetation changes, especially in N‐High, while radiation has a negligible impact on LAI. In N‐Mid and S‐Mid, we selected SM and Tmp and ET and Tmp, respectively, to build the optimal models, without including CO_2_. Zhu et al. (2016) also validated that in these regions, climatic factors are the primary drivers of vegetation changes. In the Trop region, Zhu et al. (2016) showed that CO_2_ is the dominant factor, with climatic factors having the second‐largest impact. Therefore, the selection of ET, SM, Tmp, and CO_2_ in the Trop region for this study is well supported.

Second, in terms of model predictions, our results are largely consistent with existing studies. Under different future scenarios, LAI will continue to increase globally, with larger increases observed under higher levels of radiative forcing (Chen et al. 2022; Mahowald et al. 2016; Zhao et al. 2020). The NH, in particular, is expected to be a hotspot for vegetation greening (Mahowald et al. 2016; Nolan et al. 2018; Zhao et al. 2020). Chen et al. (2022) also indicated that the future greening hotspot will be concentrated in the NH, especially in northeastern Asia. In contrast, the S‐Mid region shows evidence of browning, and the greening rate does not significantly increase with higher radiative forcing levels (Chen et al. 2022; Zhao et al. 2020).

### 5.4 The Impacts of Human Activities and Vapor Pressure Deficit on Vegetation Changes

Human activities have a significant impact on vegetation (Piao et al. 2020). Relevant studies have shown that China and India have played a leading role in global greening through land management. Specifically, China has contributed 25% of global vegetation greening through afforestation and cropland expansion, while India's greening has been primarily driven by cropland expansion (Chen et al. 2019).

In this study, the selected experiment IDs from CMIP6 include historical, SSP126, SSP245, SSP370, and SSP585. The historical dataset covers the period from 1982 to 2014, incorporating various known natural and anthropogenic factors to validate the capability of climate models in simulating past climate changes. Meanwhile, SSP126, SSP245, SSP370, and SSP585 represent future projections from 2014 to 2100 under different emission scenarios, considering both natural and anthropogenic influences (Eyring et al. 2016). Therefore, to assess the impact of human activities, we employ data from the historical, SSP126, SSP245, SSP370, and SSP585 experiments, incorporating anthropogenic factors such as greenhouse gases, aerosols, and land‐use changes to model vegetation dynamics.

The vapor pressure deficit (VPD) also affects vegetation changes. Relevant studies have shown that an increase in VPD and a decrease in SM have triggered 37.62% of compound drought events globally, leading to vegetation browning (Liu et al. 2023). Additionally, extreme climate events, solar radiation, and sunshine duration also have significant impacts on vegetation growth and ecosystem functions (Chen et al. 2024; Dai et al. 2023; Wang et al. 2024a; Wang et al. 2024b).

Due to the limitations of the CMIP6 datasets, VPD is not considered one of the impact factors. In future work, we will further explore vegetation responses to a wider range of climate factors, such as VPD, solar radiation, and sunshine duration.

## 6 Conclusions

This study first proposed new optimal combined models to project the future global vegetation using five climatic factors derived from two CMIP 6 datasets and their averaged datasets (DISO‐W, MME), and six AI algorithms based on CCHZ‐DISO. These new optimal combined models fully include the spatial heterogeneities of the climate factors, AI models, and all their combinations, which are not considered in other literature. Against the other traditional AI models, our new optimal combined models have the best performance in simulating the vegetation variations, which is very important to obtain a reliable projection. The main findings are displayed as follows:

1. From 1982 to 2014, global vegetation showed an overall greening trend, with 32.41% of the vegetated area experiencing significant greening. Globally, LAI increased at a rate of 0.072 m^2^ m^−2^ year^−1^.
2. Among the four datasets, CESM2‐WACCM was selected in the optimal combined models for the mid‐latitude regions, and CanESM5 was chosen for the remaining regions.
3. Globally, SM and CO_2_ are the key factors influencing vegetation. Tmp is the dominant factor driving vegetation changes, especially in N‐High. Except for S‐Mid, SM also has a substantial influence in other regions, while CO_2_ primarily affects vegetation in the Trop region. There is considerable variability among the models built with different combinations of climatic factors, and more factors do not necessarily lead to better models. Model stability improves when three or more climatic factors are included in the combination, and the optimal models for each region involve 2–4 climatic factors.
4. The performance of different algorithms varies significantly across regions. The optimal algorithms selected for the best models are RF for the Global and NH, LSTM for S‐Mid, and LR for the Trop region. CNN and Transformer perform poorly in all regions, and all the algorithms lose their performance in the Trop region.
5. In the future, as radiative forcing levels increase, global LAI is projected to rise accordingly, with the NH emerging as a hotspot for greening. Under low‐emission scenarios, N‐High will be the key region for vegetation growth, while under high‐emission scenarios, N‐Mid will take the lead. Across all SSP scenarios, the N‐High region exhibits the largest increase in the greening rate in the future, relative to 1982–2014, with a range of −0.49 to 2.25 times. After 2061, the greening rate of LAI is expected to slow in all regions, with some regions even experiencing browning.

The new optimal combined models developed in our study comprehensively consider the spatial heterogeneities of the climate factors, AI models, and all their combinations, which enhance our understanding of future water, carbon, and energy cycles under climate change, improving our overall comprehension of climate impacts. Moreover, this approach offers a new method for projecting other geographic and environmental factors in geography, meteorology, and other subjects.

## Author Contributions

**Hao Zhang:** conceptualization, data curation, formal analysis, investigation, methodology, resources, software, supervision, validation, visualization, writing – original draft. **Zengyun Hu:** funding acquisition, investigation, methodology, writing – original draft, writing – review and editing. **Xi Chen:** project administration, writing – review and editing. **Jianfeng Li:** writing – original draft, writing – review and editing. **Qianqian Zhang:** software. **Xiaowei Zheng:** visualization.

## Conflicts of Interest

The authors declare no conflicts of interest.

## Supporting information


Data S1.

## Data Availability

The code that supports the findings of this study are openly available in Zenodo at https://zenodo.org/records/14954831. Global Inventory Modeling and Mapping Studies (GIMMS) Leaf Area Index (LAI) data were obtained from NOAA National Centers for Environmental Information at https://doi.org/10.7289/V5TT4P69. Global LAnd Surface Satellite (GLASS) Leaf Area Index (LAI) data were obtained from http://www.glass.umd.edu/Download.html (Version 40). The GLOBMAP LAI datasets were obtained from the USDA Ag Data Commons via Zenodo at https://zenodo.org/records/4700264. The CMIP6 datasets were obtained from Earth System Grid Federation at http://doi.org/10.22033/ESGF/CMIP6.3610 and http://doi.org/10.22033/ESGF/CMIP6.10071.
