# Supplementary material for: Global Greening Major Contributed by Climate Change With More Than Two Times Rate Against the History Period During the 21th Century
Source: Glob Chang Biol. 2025 Mar 11;31(3):e70126. doi: 10.1111/gcb.70126 (PMC11897688; doi:10.1111/gcb.70126)
Supplement: Supplementary file 1 — Data S1. [file GCB-31-e70126-s001.docx]

**Global Greening major contributed by climate change with more than two times rate against the history period during the 21th Century**

Hao Zhang^1,4a^, Zengyun Hu^2a*^, Xi Chen^3*^, Jianfeng Li^5^, Qianqian Zhang^2^, Xiaowei Zheng^2^

^1^State Key Laboratory of Ecological Safety and Sustainable Development in Arid Lands, Xinjiang Institute of Ecology and Geography, Chinese Academy of Sciences, Urumqi, Xinjiang 830011, China

^2^ School of Global Health, Chinese Center for Tropical Diseases Research, Shanghai Jiao Tong University School of Medicine, Shanghai 200025, China

^3^ College of Geoinformatics, Zhejiang University of Technology, Hangzhou 310014, China

^4^University of Chinese Academy of Sciences, Beijing 100049, China

^5^[The Chinese University of Hong Kong](https://www.cuhk.edu.hk/chinese/), Hong Kong 999077, China

***Corresponding author:**

**Zengyun Hu, Email: [hzyhjq@sjtu.edu.cn](mailto:hzyhjq@sjtu.edu.cn)**

**Xi Chen, Email: chenxi@zjut.edu.cn**

**^a^Hao Zhang and Zengyun Hu should be considered joint first author**

**Supplementary Information**

**S1. Linear Regression**

As a fundamental machine learning method, Linear Regression (LR) is a classic supervised learning algorithm used to model linear relationships. It predicts outcomes using one or more easily measurable variables. The mathematical expression of the linear regression model is:

$$y=\beta_{0}+\beta_{1}x_{1}+\beta_{2}x_{2}+\beta_{3}x_{3}+\ldots+\beta_{n}x_{n}+\varepsilon$$

where $y$ is the predicted variable, also known as the dependent variable, $x_{1},x_{2},x_{3},\ldots{, x}_{n}$ is the independent variable, $\beta_{0}$ is the intercept, representing the predicted value when the independent variable is zero, $\beta_{1},\beta_{2}{,\beta}_{3},...,\beta_{n}$ is the regression coefficient, and $\varepsilon$ is the error term, capturing the random discrepancies between the model predictions and actual observations.

Due to its simplicity and ease of use, linear regression has been widely applied (Hu et al., 2014, 2019, 2022; Peng et al., 2024; Song et al., 2023; Zhang et al., 2024).

**S2. Support Vector Regression**

Support Vector Regression (SVR) is an extension of support vector machines (SVM) for regression analysis, designed to solve regression problems (Drucker et al., 1996). Its objective is to keep the model as simple as possible while closely approximating the training function within an allowable margin of error. SVR has been proven to be an effective tool for function estimation (Awad & Khanna, 2015). As a supervised learning method and one of the classic machine learning algorithms, SVR uses a symmetric loss function that penalizes overestimation and underestimation equally. One of its primary advantages is that its computational complexity does not depend on the dimensionality of the input space. Moreover, it has excellent generalization capabilities and high predictive accuracy. SVR has been widely applied in geographic studies for various prediction tasks (Chen et al., 2010; Shamshirband et al., 2020; Yu et al., 2022).

**S3. Random Forest**

Random Forest (RF) is one of the most widely used machine learning algorithms, categorized under supervised learning (Breiman, 2001). Since its introduction in 2001, RF has been extensively developed and applied in the field of geography (Giamalaki et al., 2022; Jozwicki et al., 2022; Mao & Sorteberg, 2020; Su et al., 2020; Wang et al., 2021; Zhang et al., 2024).

RF is a decision tree-based algorithm that improves model accuracy by combining the predictions of multiple decision trees. It enhances model diversity and reduces overfitting issues commonly associated with a single decision tree. RF can be applied to both classification problems and regression tasks, where it predicts continuous values by averaging the predictions from all the decision trees to generate the final regression result. It performs well with high-dimensional data, effectively reducing variance and improving the predictive performance of the model (Breiman, 2001).

**S4. Convolutional Neural Network**

Convolutional Neural Network (CNN) is a deep learning algorithm designed to handle data with network topologies and is categorized under supervised learning (LeCun et al., 1989). It has demonstrated powerful performance in the fields of computer vision and image processing (Zhang et al., 2018), excelling at extracting mid- to high-level abstract features from raw images through alternating convolutional and pooling layers, which progressively reduce the spatial size of the feature maps (Zhu et al., 2017). The main components of CNN include convolutional layers, pooling layers, activation functions, fully connected layers, and normalization layers, with the convolutional layer being the core structure responsible for extracting local features from the input data (Elbeltagi et al., 2024). CNN has been widely applied in remote sensing image recognition (Wang et al., 2020; Zhang et al., 2018), geographic feature prediction (Elbeltagi et al., 2024), and atmospheric pollution forecasting (Yan et al., 2021).

**S5. Long Short-Term Memory Neural Network**

Long Short-Term Memory Neural Network (LSTM), introduced in 1997, is a classic deep learning algorithm designed to address the limitations of recurrent neural networks (RNNs) when handling long time series data (Hochreiter & Schmidhuber, 1997). By incorporating "memory cells" and "gating mechanisms," LSTM effectively avoids the issues of vanishing and exploding gradients, enabling it to capture long-term dependencies within the data more effectively (Elbeltagi et al., 2024; Hochreiter & Schmidhuber, 1997; Hochreiter, 1998). The core components of LSTM include the forget gate, input gate, and output gate. The input gate focuses on updating information from the input and identifying changes in the current cell state, while the forget gate determines which information to retain or discard (Elbeltagi et al., 2024). These gating mechanisms allow LSTM to retain important information when processing long sequences. LSTM has been widely applied in geographic prediction tasks (Chen et al., 2021; Cho & Kim, 2022; Sun et al., 2023).

**S6. Transformer**

Transformer, introduced in 2017, is a deep learning model designed for processing sequential data and was initially developed for natural language processing tasks (Vaswani et al., 2017). Unlike LSTM, it does not rely on a recursive structure for sequence processing but instead leverages a self-attention mechanism, making it more efficient when handling long-term data (Vaswani et al., 2017). The self-attention mechanism, also known as internal attention, is the core component of the Transformer. It links different positions within a single sequence to compute the sequence representation, allowing the model to allocate attention based on the relationships between different parts of the input sequence (Shen et al., 2023; Vaswani et al., 2017). Due to its exceptional ability to capture long-term dependencies and interactions between data points, Transformer has demonstrated high performance in various time series tasks, making it widely used in time series modeling (Fu et al., 2024). In the field of geography, it has been primarily applied to image recognition and time series forecasting problems (Demiray et al., 2024; Fang et al., 2024; Fu et al., 2024; Shen et al., 2023).

**S7. Analysis of LAI Browning in 2000-2001**

In this study, browning was observed across all regions except for S-Mid during the 2000-2001 period (Figure 4). However, related studies suggest that globally, this period was marked by greening. For example, Zhu (2016) analyzed multiple LAI datasets, all showing an increasing trend during 2000-2001. Other studies also support this conclusion (Li et al., 2024; Piao et al., 2020).

In terms of the data itself, the LAI used in this study is an ensemble average product derived from three datasets: GIMMS, GLOBMAP, and GLASS. The GIMMS LAI is derived from NDVI using an artificial neural network (ANN) algorithm, based on NOAA-AVHRR data, providing a complete dataset from 1982 to 2014 (Vermote et al., 2019). GLOBMAP LAI is a composite of AVHRR (1981-2000) and MODIS (2000-2014) data (Liu et al., 2012). The GLASS LAI is synthesized from AVHRR LAI (1982-1999) and MODIS LAI products (2000-2014) (Liang et al., 2021; Ma & Liang, 2022; Xiao et al., 2016).

We analyzed the trends of the three LAI datasets in the Global region from 1982 to 2014 (Figure S1). During 2000-2001, GIMMS LAI showed an increasing trend, while GLOBMAP LAI exhibited a decreasing trend, and GLASS LAI experienced a sharp decline. Specifically, GLASS LAI decreased by 16% from 2000 to 2001. Furthermore, the average LAI values for GIMMS, GLOBMAP, and GLASS during 1982-2014 were 1.39, 1.28, and 1.05 m^2^m^-2^, respectively, with GLASS LAI having the lowest average. This suggests that the browning observed in the ensemble LAI during 2000-2001 was primarily driven by the GLASS LAI dataset.

However, this does not affect the representation of global vegetation change by the ensemble average LAI from 1982 to 2014. In the Global region, our study shows a greening rate of 0.072 m^2^m^-2^year^-1^ from 1982 to 2014. Zhu (2016) reported a global greening rate of 0.068 ± 0.045 m^2^m^-2^year^-1^ between 1982 and 2009, indicating that our findings are generally consistent. While the three datasets show alignment in overall global trends, differences in their derivation methods and data sources lead to significant uncertainties in specific regions. Despite these uncertainties, the three datasets still hold substantial research significance (Zhu et al., 2016).

**References**

Awad, M., & Khanna, R. (2015). Support Vector Regression. In M. Awad & R. Khanna (Eds.), *Efficient Learning Machines: Theories, Concepts, and Applications for Engineers and System Designers* (pp. 67–80). Apress. https://doi.org/10.1007/978-1-4302-5990-9_4

Breiman, L. (2001). Random Forests. *Machine Learning*, *45*(1), 5–32. https://doi.org/10.1023/A:1010933404324

Chen, S.-T., Yu, P.-S., & Tang, Y.-H. (2010). Statistical downscaling of daily precipitation using support vector machines and multivariate analysis. *Journal of Hydrology*, *385*(1), 13–22. https://doi.org/10.1016/j.jhydrol.2010.01.021

Chen, Z., Liu, H., Xu, C., Wu, X., Liang, B., Cao, J., & Chen, D. (2021). Modeling vegetation greenness and its climate sensitivity with deep-learning technology. *Ecology and Evolution*, *11*(12), 7335–7345. https://doi.org/10.1002/ece3.7564

Cho, K., & Kim, Y. (2022). Improving streamflow prediction in the WRF-Hydro model with LSTM networks. *Journal of Hydrology*, *605*, 127297. https://doi.org/10.1016/j.jhydrol.2021.127297

Demiray, B. Z., Sit, M., Mermer, O., & Demir, I. (2024). Enhancing hydrological modeling with transformers: A case study for 24-h streamflow prediction. *Water Science and Technology*, *89*(9), 2326–2341. https://doi.org/10.2166/wst.2024.110

Drucker, H., Burges, C. J. C., Kaufman, L., Smola, A., & Vapnik, V. (1996). Support vector regression machines. *Proceedings of the 9th International Conference on Neural Information Processing Systems*, 155–161.

Elbeltagi, A., Srivastava, A., Ehsan, M., Sharma, G., Yu, J., Khadke, L., Gautam, V. K., Awad, A., & Jinsong, D. (2024). Advanced stacked integration method for forecasting long-term drought severity: CNN with machine learning models. *Journal of Hydrology: Regional Studies*, *53*, 101759. https://doi.org/10.1016/j.ejrh.2024.101759

Fang, J., Yang, L., Wen, X., Yu, H., Li, W., Adamowski, J. F., & Barzegar, R. (2024). Ensemble learning using multivariate variational mode decomposition based on the Transformer for multi-step-ahead streamflow forecasting. *Journal of Hydrology*, *636*, 131275. https://doi.org/10.1016/j.jhydrol.2024.131275

Fu, Y., Song, J., Guo, J., Fu, Y., & Cai, Y. (2024). Prediction and analysis of sea surface temperature based on LSTM-transformer model. *Regional Studies in Marine Science*, *78*, 103726. https://doi.org/10.1016/j.rsma.2024.103726

Giamalaki, K., Beaulieu, C., & Prochaska, J. X. (2022). Assessing Predictability of Marine Heatwaves With Random Forests. *Geophysical Research Letters*, *49*(23), e2022GL099069. https://doi.org/10.1029/2022GL099069

Hochreiter, S., & Schmidhuber, J. (1997). Long Short-Term Memory. *Neural Computation*, *9*(8), 1735–1780. https://doi.org/10.1162/neco.1997.9.8.1735

Hu, Z., Chen, X., Chen, D., Li, J., Wang, S., Zhou, Q., Yin, G., & Guo, M. (2019). “Dry gets drier, wet gets wetter”: A case study over the arid regions of central Asia. *International Journal of Climatology*, *39*(2), 1072–1091. https://doi.org/10.1002/joc.5863

Hu, Z., Chen, X., Zhou, Q., Yin, G., & Liu, J. (2022). Dynamical variations of the terrestrial water cycle components and the influences of the climate factors over the Aral Sea Basin through multiple datasets. *Journal of Hydrology*, *604*, 127270. https://doi.org/10.1016/j.jhydrol.2021.127270

Hu, Z., Zhang, C., Hu, Q., & Tian, H. (2014). Temperature Changes in Central Asia from 1979 to 2011 Based on Multiple Datasets. *Journal of Climate*, *27*(3), 1143–1167. https://doi.org/10.1175/JCLI-D-13-00064.1

Jozwicki, D., Sharma, P., Mann, I., & Hoppe, U.-P. (2022). Segmentation of PMSE Data Using Random Forests. *Remote Sensing*, *14*(13), Article 13. https://doi.org/10.3390/rs14132976

LeCun, Y., Boser, B., Denker, J. S., Henderson, D., Howard, R. E., Hubbard, W., & Jackel, L. D. (1989). Backpropagation Applied to Handwritten Zip Code Recognition. *Neural Computation*, *1*(4), 541–551. https://doi.org/10.1162/neco.1989.1.4.541

Li, X., Wang, K., Huntingford, C., Zhu, Z., Peñuelas, J., Myneni, R. B., & Piao, S. (2024). Vegetation greenness in 2023. *Nature Reviews Earth & Environment*, *5*(4), 241–243. https://doi.org/10.1038/s43017-024-00543-z

Liang, S., Cheng, J., Jia, K., Jiang, B., Liu, Q., Xiao, Z., Yao, Y., Yuan, W., Zhang, X., Zhao, X., & Zhou, J. (2021). The Global Land Surface Satellite (GLASS) Product Suite. *Bulletin of the American Meteorological Society*, *102*(2), E323–E337. https://doi.org/10.1175/BAMS-D-18-0341.1

Liu, Y., Liu, R., & Chen, J. M. (2012). Retrospective retrieval of long-term consistent global leaf area index (1981–2011) from combined AVHRR and MODIS data. *Journal of Geophysical Research: Biogeosciences*, *117*(G4). https://doi.org/10.1029/2012JG002084

Ma, H., & Liang, S. (2022). Development of the GLASS 250-m leaf area index product (version 6) from MODIS data using the bidirectional LSTM deep learning model. *Remote Sensing of Environment*, *273*, 112985. https://doi.org/10.1016/j.rse.2022.112985

Mao, Y., & Sorteberg, A. (2020). *Improving Radar-Based Precipitation Nowcasts with Machine Learning Using an Approach Based on Random Forest*. https://doi.org/10.1175/WAF-D-20-0080.1

Peng, Y., Zhang, H., Zhang, Z., Tang, B., Shen, D., Yin, G., Li, Y., Chen, X., Hu, Z., & Habib Nazrollozoda, S. (2024). Future challenges of terrestrial water storage over the arid regions of Central Asia. *International Journal of Applied Earth Observation and Geoinformation*, *132*, 104026. https://doi.org/10.1016/j.jag.2024.104026

Piao, S., Wang, X., Park, T., Chen, C., Lian, X., He, Y., Bjerke, J. W., Chen, A., Ciais, P., Tømmervik, H., Nemani, R. R., & Myneni, R. B. (2020). Characteristics, drivers and feedbacks of global greening. *Nature Reviews Earth & Environment*, *1*(1), Article 1. https://doi.org/10.1038/s43017-019-0001-x

Shamshirband, S., Hashemi, S., Salimi, H., Samadianfard, S., Asadi, E., Shadkani, S., Kargar, K., Mosavi, A., Nabipour, N., & Chau, K.-W. (2020). Predicting Standardized Streamflow index for hydrological drought using machine learning models. *Engineering Applications of Computational Fluid Mechanics*, *14*(1), 339–350. https://doi.org/10.1080/19942060.2020.1715844

Shen, L., Su, H., Li, Z., Jia, C., & Yang, R. (2023). Self-Attention-Based Transformer for Nonlinear Maneuvering Target Tracking. *IEEE Transactions on Geoscience and Remote Sensing*, *61*, 1–13. IEEE Transactions on Geoscience and Remote Sensing. https://doi.org/10.1109/TGRS.2023.3312314

Song, S., Chen, X., Liu, T., Zan, C., Hu, Z., Huang, S., De Maeyer, P., Wang, M., & Sun, Y. (2023). Indicator-based assessments of the coupling coordination degree and correlations of water-energy-food-ecology nexus in Uzbekistan. *Journal of Environmental Management*, *345*, 118674. https://doi.org/10.1016/j.jenvman.2023.118674

Su, H., Shen, W., Wang, J., Ali, A., & Li, M. (2020). Machine learning and geostatistical approaches for estimating aboveground biomass in Chinese subtropical forests. *Forest Ecosystems*, *7*(1), 64. https://doi.org/10.1186/s40663-020-00276-7

Sun, K., Hu, L., Sun, J., & Cao, X. (2023). Enhancing groundwater level prediction accuracy at a daily scale through combined machine learning and physics-based modeling. *Journal of Hydrology: Regional Studies*, *50*, 101577. https://doi.org/10.1016/j.ejrh.2023.101577

Sun, Y., Lao, D., Ruan, Y., Huang, C., & Xin, Q. (2023). A Deep Learning-Based Approach to Predict Large-Scale Dynamics of Normalized Difference Vegetation Index for the Monitoring of Vegetation Activities and Stresses Using Meteorological Data. *Sustainability*, *15*(8), Article 8. https://doi.org/10.3390/su15086632

Vaswani, A., Shazeer, N., Parmar, N., Uszkoreit, J., Jones, L., Gomez, A. N., Kaiser, Ł. ukasz, & Polosukhin, I. (2017). Attention is All you Need. *Advances in Neural Information Processing Systems*, *30*. https://proceedings.neurips.cc/paper_files/paper/2017/hash/3f5ee243547dee91fbd053c1c4a845aa-Abstract.html

Vermote, Eric; NOAA CDR Program. (2019): NOAA Climate Data Record (CDR) of AVHRR Leaf Area Index (LAI) and Fraction of Absorbed Photosynthetically Active Radiation (FAPAR), Version 5. NOAA National Centers for Environmental Information. https://doi.org/10.7289/V5TT4P69

Wang, H., Seaborn, T., Wang, Z., Caudill, C. C., & Link, T. E. (2021). Modeling tree canopy height using machine learning over mixed vegetation landscapes. *International Journal of Applied Earth Observation and Geoinformation*, *101*, 102353. https://doi.org/10.1016/j.jag.2021.102353

Wang, Y., Fang, Z., Hong, H., & Peng, L. (2020). Flood susceptibility mapping using convolutional neural network frameworks. *Journal of Hydrology*, *582*, 124482. https://doi.org/10.1016/j.jhydrol.2019.124482

Xiao, Z., Liang, S., Wang, J., Xiang, Y., Zhao, X., & Song, J. (2016). Long-Time-Series Global Land Surface Satellite Leaf Area Index Product Derived From MODIS and AVHRR Surface Reflectance. *IEEE Transactions on Geoscience and Remote Sensing*, *54*(9), 5301–5318. IEEE Transactions on Geoscience and Remote Sensing. https://doi.org/10.1109/TGRS.2016.2560522

Yan, R., Liao, J., Yang, J., Sun, W., Nong, M., & Li, F. (2021). Multi-hour and multi-site air quality index forecasting in Beijing using CNN, LSTM, CNN-LSTM, and spatiotemporal clustering. *Expert Systems with Applications*, *169*, 114513. https://doi.org/10.1016/j.eswa.2020.114513

Yu, J.-W., Kim, J.-S., Jong, Y.-C., Li, X., & Ryang, G.-I. (2022). Forecasting chlorophyll-a concentration using empirical wavelet transform and support vector regression. *Journal of Forecasting*, *41*(8), 1691–1700. https://doi.org/10.1002/for.2890

Zhang, C., Sargent, I., Pan, X., Li, H., Gardiner, A., Hare, J., & Atkinson, P. M. (2018). An object-based convolutional neural network (OCNN) for urban land use classification. *Remote Sensing of Environment*, *216*, 57–70. https://doi.org/10.1016/j.rse.2018.06.034

Zhang, H., Hu, Z., Zhang, Z., Li, Y., Song, S., & Chen, X. (2024). How does vegetation change under the warm–wet tendency across Xinjiang, China? *International Journal of Applied Earth Observation and Geoinformation*, *127*, 103664. https://doi.org/10.1016/j.jag.2024.103664

Zhang, X., Shen, H., Huang, T., Wu, Y., Guo, B., Liu, Z., Luo, H., Tang, J., Zhou, H., Wang, L., Xu, W., & Ou, G. (2024). Improved random forest algorithms for increasing the accuracy of forest aboveground biomass estimation using Sentinel-2 imagery. *Ecological Indicators*, *159*, 111752. https://doi.org/10.1016/j.ecolind.2024.111752

Zhu, X. X., Tuia, D., Mou, L., Xia, G.-S., Zhang, L., Xu, F., & Fraundorfer, F. (2017). Deep Learning in Remote Sensing: A Comprehensive Review and List of Resources. *IEEE Geoscience and Remote Sensing Magazine*, *5*(4), 8–36. IEEE Geoscience and Remote Sensing Magazine. https://doi.org/10.1109/MGRS.2017.2762307

Zhu, Z., Piao, S., Myneni, R. B., Huang, M., Zeng, Z., Canadell, J. G., Ciais, P., Sitch, S., Friedlingstein, P., Arneth, A., Cao, C., Cheng, L., Kato, E., Koven, C., Li, Y., Lian, X., Liu, Y., Liu, R., Mao, J., … Zeng, N. (2016). Greening of the Earth and its drivers. *NATURE CLIMATE CHANGE*, *6*(8), 791-+. <https://doi.org/10.1038/NCLIMATE3004>


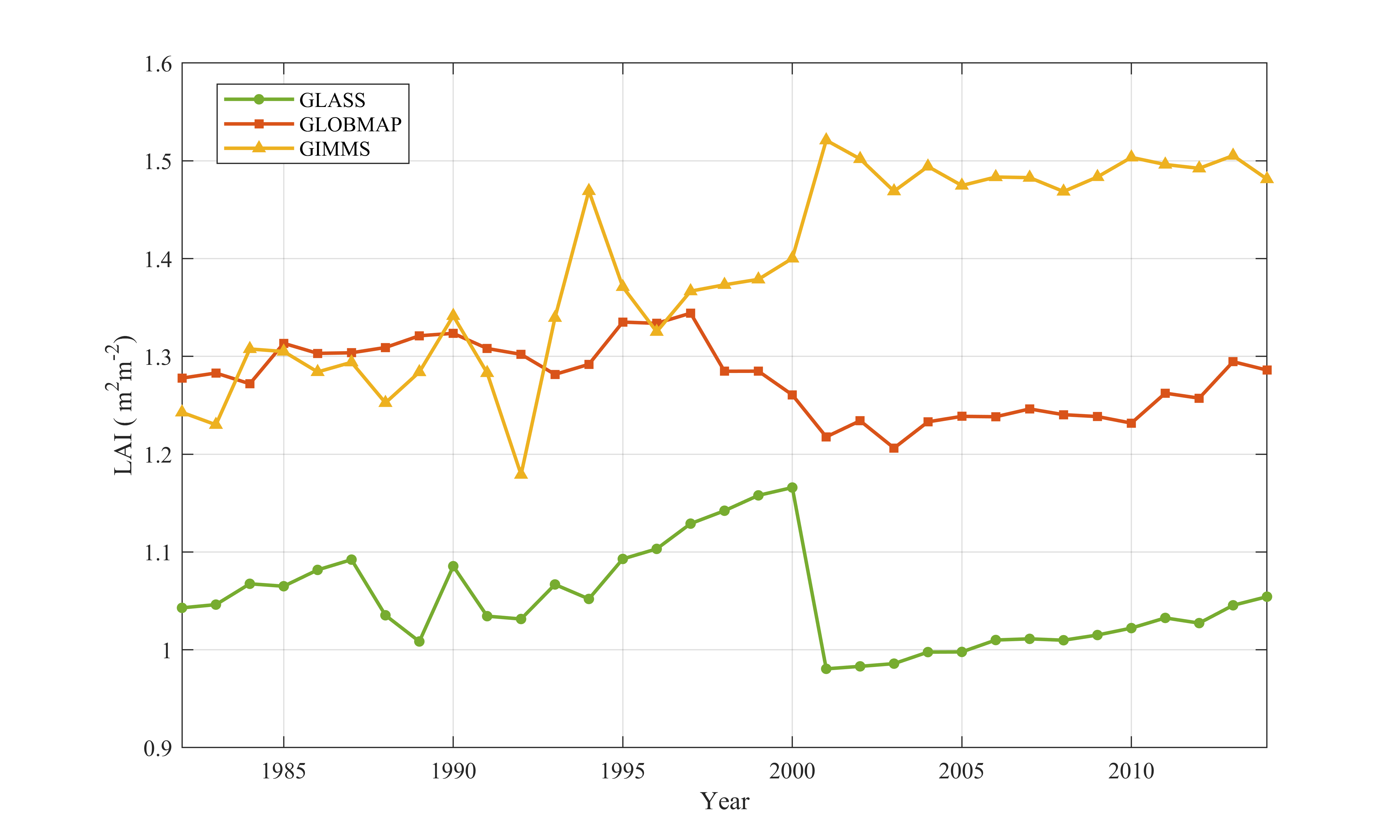


**Figure S1.** Trends of GLASS, GLOBMAP, and GIMMS LAI data from 1982 to 2014





**Figure S2.** Fitting plots of LAI for optimal models in each region. Figures S2a-e represent global, N-High, N-Mid, Trop, and S-Mid, respectively
